# Human immunodeficiency virus type 1 specific cytotoxic T lymphocyte responses in Chinese infected with HIV-1 B'/C Recombinant (CRF07_BC)

**DOI:** 10.1186/1742-4690-4-62

**Published:** 2007-08-30

**Authors:** Jianping Chen, Kunxue Hong, Mingming Jia, Hongwei Liu, Yuanzhi Zhang, Sha Liu, Xiaoqing Zhang, Hongjing Zhao, Hong Peng, Pengfei Ma, Hui Xing, Yuhua Ruan, Katie L Williams, Xu G Yu, Marcus Altfeld, Bruce D Walker, Yiming Shao

**Affiliations:** 1 State Key Laboratory for Infectious Disease Control and Prevention, National Center for AIDS/STD Control and Prevention, Chinese Center for Disease Control and Prevention, Beijing 100050, China; 2 Xinjiang Center for Disease Control and Prevention, Urumuqi, Xinjiang 830011, China; 3 Partners AIDS Research Center, Massachusetts General Hospital, and Division of AIDS, Harvard Medical School, Boston, MA 02114, USA

## Abstract

**Background:**

The characterization of HIV-1-specific T cell responses in people infected with locally circulating HIV-1 strain will facilitate the development of HIV-1 vaccine. Sixty intravenous drug users infected with HIV-1 circulating recombinant form 07_BC (CRF07_BC), which has been spreading rapidly in western China from north to south, were recruited from Xinjiang, China to assess the HIV-1-specific T cell responses at single peptide level with overlapping peptides (OLP) covering the whole concensus clades B and C proteome.

**Results:**

The median of the total magnitude and total number of OLPs recognized by CTL responses were 10925 SFC/million PBMC and 25 OLPs, respectively, when tested by clade C peptides, which was significantly higher than when tested by clade B peptides. The immunodominant regions, which cover 14% (58/413) of the HIV-1 proteome, are widely distributed throughout the HIV-1 proteome except in Tat, Vpu and Pol-PR, with Gag, Pol-RT, Pol-Int and Nef being most frequently targeted. The subdominant epitopes are mostly located in p24, Nef, integrase, Vpr and Vif. Of the responses directed to clade C OLPs, 61.75% (972/1574) can be observed when tested with corresponding clade B OLPs. However, Pol-PR and Vpu tend to be targeted in the clade B sequence rather than the clade C sequence, which is in line with the recombinant pattern of CRF07_BC. Stronger and broader CTL responses in subjects with CD4 cell counts ranging from 200 to 400/mm^3 ^were observed when compared to those with less than 200/mm^3 ^or more than 400/mm^3^, though there have been no significant correlations identified between the accumulative CTL responses or overall breadth and CD4 cell count or plasma viral load.

**Conclusion:**

This is the first study conducted to comprehensively address T cell responses in Chinese subjects infected with HIV-1 CRF07_BC in which subtle differences in cross-reactivity were observed, though similar patterns of overall immune responses were demonstrated with clade B infected populations. The immunodominant regions identified in this population can facilitate future HIV-1 vaccine development in China.

## Background

HIV-1 specific cytotoxic T lymphocyte (CTL) responses play pivotal roles in driving HIV-1 evolution [1-3] and controlling viral infection [4,5]. Immune escape through mutations within CTL epitopes is rapidly accumulated in the HIV-1 genome [1-3], indicating the existence of a strong selective pressure of immune responses on HIV-1 evolution. Dramatic declines of initial peak viremia to viral set point are observed in acute HIV-1 infection with the emergence of CTL responses[4] and strong CTL responses are detected in long-term nonprogressors with chronic HIV-1 infection [5]. At the population level, the correlation between HIV-1-specific, especially Gag-specific, CTL responses and immune control have been observed and confirmed in independent cohort studies [6-8]. Therefore, prophylactic and therapeutic HIV-1-specific vaccine candidates aiming at eliciting potent HIV-1-specific T cell responses are increasingly being tested in pre-clinical and clinical trials.

The measurement of CTL responses using peptide sets covering the whole HIV-1 expressed genome has been employed in many previous studies and covering multiple ethnicities including African, Caucasian, and Hispanic populations [9-13]. From these studies, consistent CTL targeting of immunodominant regions in the HIV-1 proteome has been recorded [10] and a high degree of inter-clade cross-reactivity of HIV-1-specific T cell responses at the single peptide level has been observed [14]. However, the high genetic diversity of HIV-1, which is driven by high mutation rates and inter-subtype recombination rates, is a major obstacle in the successful immune containment of viral infection and therefore the design of an HIV-1 vaccine [15]. Previous studies have mainly focused on populations infected with HIV-1 clades B and clade C, which are found circulating widely throughout the world. However, the characterization of CTL responses in people infected with locally circulating HIV-1 has yet to be thoroughly conducted.

As a developing and most populous country, China is currently facing great challenges of the HIV-1 epidemic and 650,000 people are estimated to be living with HIV/AIDS in China by the end of 2005[16]. The epidemic is mainly driven by the wide spread of clade B' in former plasma donors and B'/C recombinant (Circulating Recombinant Form 07_BC, CRF07_BC) in intravenous drug users (IDUs)[17]. The CRF07_BC, showing mosaic pattern in its genome with a clade C backbone inserted by several clade Thai B fragments in Gag, Pol, Env and accessory genes[18,19] has been spreading rapidly in western China from north to south [20-22]. In this study, we assessed the profile of CTL responses in a Chinese IDU population infected with HIV-1 CRF07_BC. By employing ELISPOT using 2 sets of peptides covering the consensus clades B and C HIV-1 whole expressed genome, we have evaluated the breadth, magnitude, immunodominance and cross-recognition of CTL responses in this CRF07_BC infected Chinese population. The correlation between CTL responses and the containment of viral replication was also explored.

## Results

Previous studies have shown that HIV-1 clade C infection may result in decreased disease progression when compared to clade B infection, which also correlates with the rapid outspread of clade C strains in South Africa and the Indian subcontinent [23-25]. To obtain new insight on this issue, here we focused on the immunological responses of a Chinese population infected with CRF07_BC, a form of B'/C recombinant whose genome comprises of a clade C backbone and several insertions derived from Thai B[18,21,22].

### ELISPOT measured the CD8 CTL responses

We compared the cumulative HIV-1 specific T cell responses, which were derived from the addition of individual positive responses in ELISPOT assays at the single peptide level and in ICS assays using peptide pools. The data indicate that the ELISPOT results are very consistent with the ICS results (R = 0.96, p < 0.001). Three-color ICS was used to discriminate between the CD8 and CD4 T cell responses measured in ELISPOT and only 3 of the 60 subjects had significant CD4 T cell responses in this study.

### The magnitude and breadth of HIV-1 specific CTL responses

**Figure 1:**
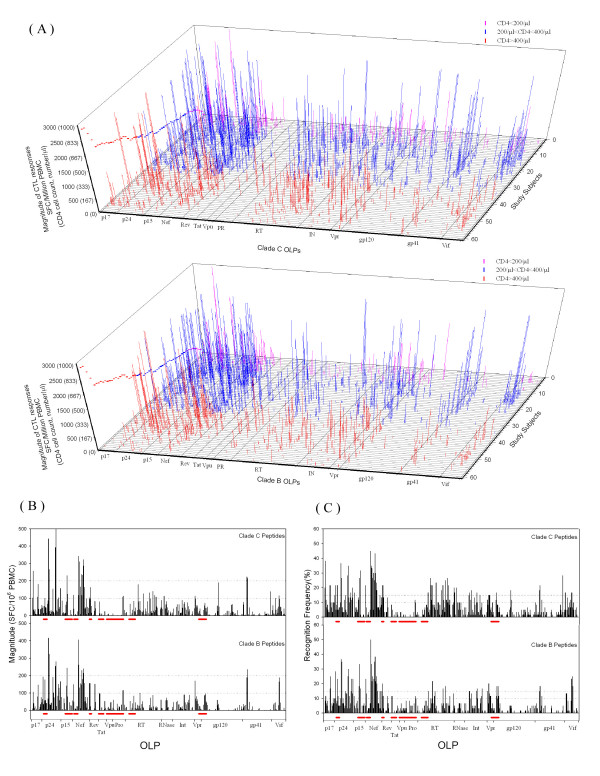
**The overall CTL responses in the study population**. (A) 3-D figures depicting individual CTL responses showing similar clustering patterns targeting clade B and Clade C peptide sets. The CD4 counts of each subject are dotted in the left of the figures. (B) The average magnitudes induced by individual peptides covering the clades B and C proteome. (C) The recognition frequency of individual peptides by the study population. Inserted clade B fragments in the CRF07_BC genome are indicated as red bars adjacent to the X-axis. Significant differences were observed when comparing the average magnitude (B) and percent of responders (C) for different peptide sets.

We examined the magnitude and frequency of recognition at the single peptide level in this study population (Figure 1, Table 1). Similar clustering patterns of CTL responses targeting the clades B and C proteome were observed (Figure 1A). However, when looking at the single peptide level, the average magnitude of CTL responses and percent of responders in the study population were significantly different between clades B and C peptide sets (p value of 0.009 and <0.001 respectively, Wilcoxon Signed Rank Test) (Figure 1B, C).

**Table 1:** Distribution of CTL Responses (breadth and strength) between HIV proteins

Protein	No. of Peptides		No. of OLP targeted at least once in the cohort (%)		No. of subjects with responses (%)		CTL strength (Mean ± SD) (SFC/10^6 ^PBMC)	
	
	B	C	B	C	B	C	B	C
Gag-p17*	*17*	*17*	*14(82.4%)*	*16(94.1%)*	*32(53.3%)*	*43(71.7%)*	*610 ± 1185*	*964 ± 1340*
*Gag-p24*	*31*	*31*	*30(96.8%)*	*30(96.8%)*	*51(85.0%)*	*52(86.7%)*	*3155 ± 3894*	*2935 ± 3390*
*Gag-p15*	*19*	*19*	*12(63.2%)*	*13(68.4%)*	*32(53.3%)*	*34(56.7%)*	*767 ± 1495*	*698 ± 1290*

Gag	67	67	56(83.6%)	59(88.1%)	55(91.7%)	54(90.0%)	*4532 ± 5512*	*4597 ± 5073*
Pol – Pro*#	*21*	*21*	*12(57.1%)*	*6(28.6%)*	*22(36.7%)*	*11(18.3%)*	*437 ± 949*	*240 ± 729*
Pol – RT*	*59*	*59*	*40(67.8%)*	*47(79.7%)*	*46(76.7%)*	*53(88.3%)*	*1047 ± 1461*	*2104 ± 2691*
Pol – Rnase*	*17*	*17*	*9(52.9%)*	*12(70.6%)*	*14(23.3%)*	*33(55.0%)*	*250 ± 1066*	*562 ± 1258*
*Pol – Int*	*36*	*36*	*27(75.0%)*	*30(83.3%)*	*38(63.3%)*	*42(70.0%)*	*864 ± 1158*	*922 ± 1228*

Pol*	133	133	88(66.2%)	95(71.4%)	52(86.7%)	58(96.7%)	2598 ± 3069	3828 ± 4090
Env-gp120*	*68*	*68*	*28(41.2%)*	*39(57.4%)*	*32(53.3%)*	*38(63.3%)*	*690 ± 1445*	*1034 ± 2062*
Env-gp41*	*46*	*46*	*17(37.0%)*	*31(67.4%)*	*29(48.3%)*	*45(75.0%)*	*649 ± 1377*	*943 ± 1389*

Env*	114	114	45(39.5%)	70(61.4%)	46(76.7%)	52(86.7%)	1339 ± 2040	1976 ± 2624
Nef	27	27	26(96.3%)	24(88.9%)	52(86.7%)	51(85.0%)	2727 ± 4305	2812 ± 4217
Rev	15	14	10(66.7%)	9(64.3%)	18(30.0%)	28(46.7%)	552 ± 1421	590 ± 1105
Tat	13	13	7(53.8%)	6(46.2%)	10(16.7%)	9(15.0%)	283 ± 1103	132 ± 496
Vpu*#	9	9	8(88.9%)	3(33.3%)	9(15.0%)	3(5.0%)	144 ± 535	24 ± 115
Vpr	11	11	10(90.9%)	11(100%)	27(45.0%)	27(45.0%)	532 ± 1070	522 ± 988
Vif	24	24	13(54.2%)	19(79.2%)	20(33.3%)	37(61.7%)	576 ± 1627	762 ± 1483

Total *	***413***	***412***	***263(63.7%)***	***296(71.8%)***	60(100%)	60(100%)	13283 ± 14223	15242 ± 14353

1. * denotes a significant difference in responses targeting clade B and C OLPs (p value < 0.05, paired t test).

2. # denotes that the CTL responses to clade B OLPs targeting protease and Vpu are significantly stronger and broader than to clade C OLPs

In Table 1, we have summarized the total and protein-specific magnitude and breadth of CTL responses measured in this study population for clades B and C peptides. Overall, we find that the responses targeting clade C proteins are stronger and broader than clade B proteins, with the exceptions being the Pol-PR and Vpu proteins. When tested with clade B peptides, the median of the total magnitude was 6,920 SFC/million PBMC with a range of 430–66,290 SFC/million PBMC, which is significantly lower than when tested by clade C peptides (median of 10925 SFC/million PBMC with the range of 210–66,130 SFC/million PBMC) (p < 0.001, paired t-test). For the median of the total number of OLPs recognized, there was also a significant difference between clades B and C peptides (median of 20.0 OLPs with the range of 4–59 OLPs by clade B peptide versus 24.5 OLPs with the range of 3–63 OLPs by clade C peptide, p < 0.001, paired t-test). When responses, within specific gene products, targeting clades B and C peptides were compared, the responses targeting Gag-p17, Env-gp120, Env-gp41, Pol-RT, Pol-RNase proteins are significantly broader and stronger for clade C (Table 1). For Rev, the difference in magnitude is of no significance, while the breadth is statistically significant (clade C > B). However, we observed that for Pol-PR and Vpu proteins, the responses targeting clade B proteins are broader and stronger than for clade C.

We have observed that up to 71.8% of the expressed HIV-1 clade C proteome can be targeted in this study population, compared with only 63.7% of the expressed HIV-1 clade B proteome. The most frequently targeted proteins are Gag-p24, Nef and Pol-RT, to which more than 85% of the subjects mounted CTL responses. However, only less than 20% of subjects recognize at least one peptide within the Vpu and Tat proteins.

### Immunodominance and cross-recognition analysis

**Figure 2:**
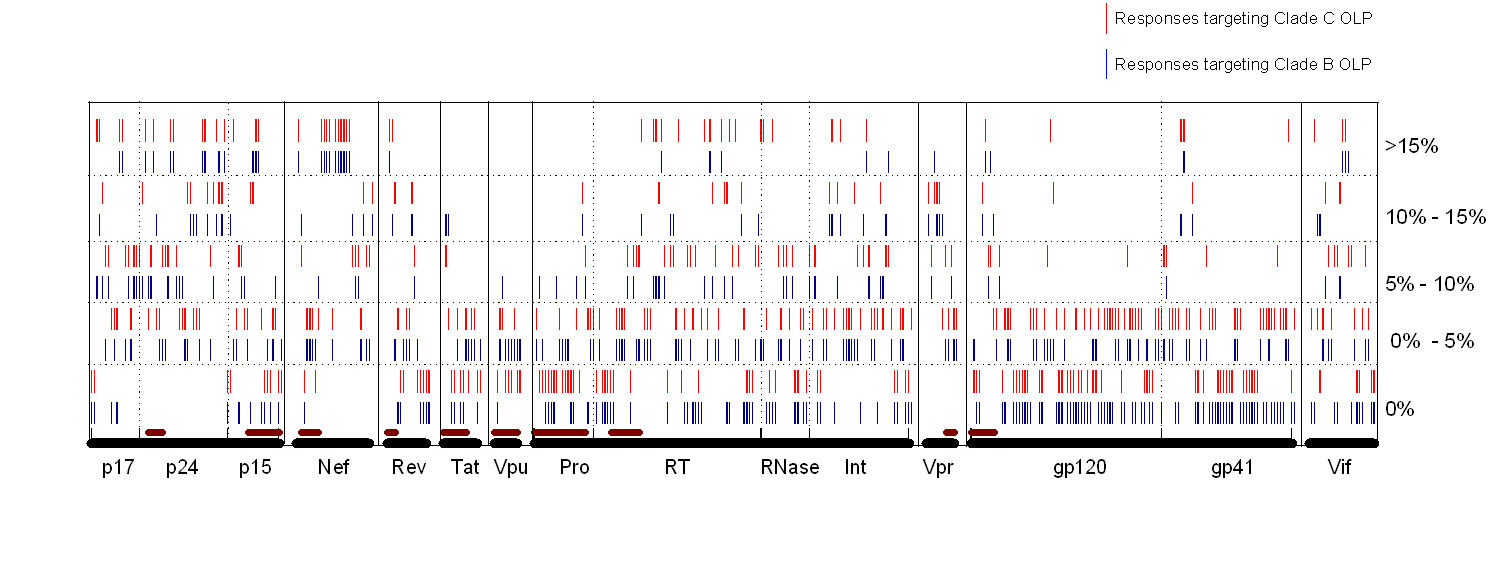
**The immunodominance and cross-reactivity analysis**. The location of immunodominant and subdominant epitopes in the HIV-1 proteome. Five classes of recognition frequency are represented in the figure, (i) recognition frequency more than 15%, (ii) more than 10% but less than 15%, (iii) more than 5% but less than 10%, (iv) more than 0% but less than 5% and (v) not recognized in the study population. Inserted clade B fragments in the CRF07_BC genome are indicated as red bars adjacent to X-axis.

We tried to identify the immunodominant region in the B'/C recombinant strains and found that there are 52 and 37 peptides from the clades C and B proteome, respectively, targeted by at least 15% of the subjects (Figure 2). These immunodominant OLPs (52 clade C and 37 clade B, total 89) cover 14% (58/413) of the HIV-1 proteome. In other words, 62 of the immunodominant OLPs detected are the corresponding clades B and C OLPs. Table 2 shows the pairs of immunodominant OLPs with different percentages of responders targeting clade B or C peptides. Notably, 26.7% of the subjects recognized the clade C OLP designated as Pol-70(located in RT protein), however with only one amino acid substitution (G359T) it is no longer detectable in the study subjects. It is also notable that there are 10 and 3 dominant epitopes identified in RT and RNase respectively when tested by clade C peptides, while only 3 and 0 identified by clade B peptides. There are no immunodominant epitopes found in Pol-PR, Tat and Vpu. The immunodominant regions are widely distributed throughout the entire genome, with Gag, Pol and Nef being the most frequently targeted. Compared to the dominant epitopes scattered within the Gag protein, those in Nef are clustered in the central region of the protein. The subdominant epitopes, targeted by more than 10% but less than 15% of the subjects, are mostly located in p24, Nef, integrase, Vpr and Vif.

**Table 2:** Sequence comparison of clade B and clade C immunodominant OLPs with different frequency of CTL Responses induced in the study population

**OLP Numbering**	**Peptide Sequences**			**Percent of Responders**		**Average Magnitude of Responders**		
	
	Clade B	Clade C	Location	Clade B	Clade C	Clade B	Clade C	**p-value**
GAG-46	TIL***K***ALGP***A***A***T***LEEMMTA	TIL***R***ALGP***G***A***S***LEEMMTA	Gag (332 – 349)	23.33%	11.67%	553	334	0.022
POL-126	***T***KIQNFRVYYRDSRDP***L***W	IKIQNFRVYYRDSRDP***I***W	Pol (933 – 950)	16.67%	10.00%	535	358	0.027
VPR-4	ELK***R***EAVRHFPRPWLHGL	ELK***Q***EAVRHFPRPWLHGL	Vpr (25 – 42)	20.00%	15.00%	405	400	0.039
ENV-8	LFCASDAKAY***DT***EVHNVW	LFCASDAKAY***EK***EVHNVW	gp160 (52 – 69)	18.33%	8.33%	525	540	N.S.
VIF-15	LIH***LY***YFDCF***SE***SAIR***N***A	LIH***MH***YFDCF***AD***SAIR***K***A	Vif (106 – 123)	18.33%	6.67%	241	773	N.S.
								
GAG-03	EKIRLRPGGKK***K***Y***R***LKHL	EKIRLRPGGKK***H***Y***M***LKHL	Gag (17 – 34)	8.33%	20.00%	128	517	0.039
GAG-04	GKK***K***Y***R***LKHLVWASREL	GKK***H***Y***M***LKHLVWASREL	Gag (25 – 41)	11.67%	38.33%	911	670	0.023
GAG-51	TNSATIMMQRGNFRNQRK	NSAILMQRSNFKGSKR	Gag (371 – 388)	5.00%	16.67%	87	520	0.016
REV-03	R***T***VR***L***IK***L***LYQSNP***L***	R***A***VR***I***IK***I***LYQSNP***Y***	Rev (14 – 28)	11.67%	21.67%	197	754	0.013
POL-43	QGWKGSPAIFQ***C***SMTKIL	QGWKGSPAIFQ***S***SMTKIL	Pol (306 – 323)	10.00%	26.67%	268	672	0.000
POL-44	IFQ***C***SMTKILEPFR***K***	IFQ***S***SMTKILEPFR***A***	Pol (314 – 328)	6.67%	21.67%	163	371	0.010
POL-61	***T***KALT***EV***VPLTEEAELEL	***A***KALT***DI***VPLTEEAELEL	Pol (441 – 458)	6.67%	18.33%	460	723	0.020
POL-70	MR***G***AHTNDVKQLTEAVQK	MR***T***AHTNDVKQLTEAVQK	Pol (512 – 529)	0.00%	26.67%	0	441	0.002
POL-72	QKIA***T***ESIVIWGKTPKF***K***	QKIA***M***ESIVIWGKTPKF***R***	Pol (528 – 545)	5.00%	21.67%	420	495	0.008
POL-81	DGAANRETK***L***GKAGYV	DGAANRETK***I***GKAGYV	Pol (598 – 613)	5.00%	20.00%	253	448	0.012
POL-82	ETK***L***GKAGYVT***NK***GRQK***V***	ETK***I***GKAGYVT***DR***GRQK***I***	Pol (604 – 621)	3.33%	16.67%	350	236	0.078
POL-85	QKTELQAI***H***LALQDSG***L***	QKTELQAI***Y***LALQDSG***S***	Pol (630 – 646)	0.00%	20.00%	0	563	0.001
POL-109	PAETGQETAYF***L***LKLAGR	PAETGQETAYF***I***LKLAGR	Pol (805 – 822)	13.33%	18.33%	264	352	N.S.
ENV-29	KV***S***F***E***PIPIHYCAPAGFA	KV***T***F***D***PIPIHYCAPAGYA	gp160 (207 – 224)	3.33%	18.33%	1120	1040	0.015
ENV-113	***Y***RAI***LH***IP*T*RIRQG***L***E***R***A	***C***RAI***RN***IP***R***RIRQG***F***E***A***A	gp160 (837 – 854)	5.00%	28.33%	110	489	0.000
VIF-3	***R***IRTW***K***SLVKHHMY***I***S***KK***	***K***IRTW***N***SLVKHHMY***V***S***RR***	Vif (17 – 34)	0.00%	16.67%	0	354	0.007

P values were for comparison of paired t-test and p = 0.05 is considered as statistic significance level.

The peptide location were indicated in the table in the reference of HIV-1 strain Hxb2.

**Figure 3:**
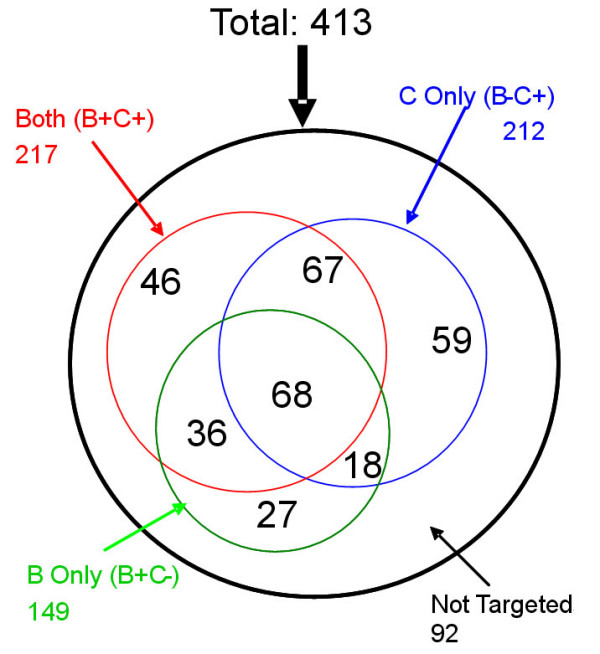
**Area-proportional Venn diagram of cross recognition**. The expressed whole genome of HIV-1 clade B or clade C were digested as 413 overlapping peptides and the two sets of peptides were tested in ELISPOT assay for each subject enrolled in this study. Cross-recognition of CTL responses to clade B and Clade C peptides were assessed by the classification of (i) both B and C peptides not recognized, (ii) both B and C peptides recognized by at least one subject, (iii) only C peptides recognized by at least one subject and (iv) only B peptides recognized by at least one subject.

High cross-recognition of HIV-1 specific CTL responses was observed in this study. When looking at the CTL recognition frequency at the single peptide level, along with the distribution of immunodominant OLPs, the profile of cross-recognition between clade B and C peptides can clearly be seen (Figure 2). To further assess the cross-recognition of CTL responses to clades B and C peptides, the two peptide sets were classified into the following categories, (i) both B and C peptides not recognized, (ii) both B and C peptides recognized by at least one subject, (iii) only C peptides recognized by at least one subject and (iv) only B peptides recognized by at least one subject. The results are represented with a Venn diagram (Figure 3) and about 22% of the corresponding OLPs (92/413) derived from both the clades B and C proteome are not targeted by CTL. Of the remaining OLPs, more than 68% (219/321) can be cross-recognized. We also analyzed the cross-recognition by looking at the total CTL responses detected by clades B and C peptide sets. There are 1352 responses observed when applying clade B OLPs, and 1574 responses to clade C OLPs. Of the responses directed to clade C OLPs, 61.75% (972/1574) can be observed when tested with corresponding clade B OLPs.

### Correlation of CTL responses with immune control of HIV-1 infection

**Figure 4:**
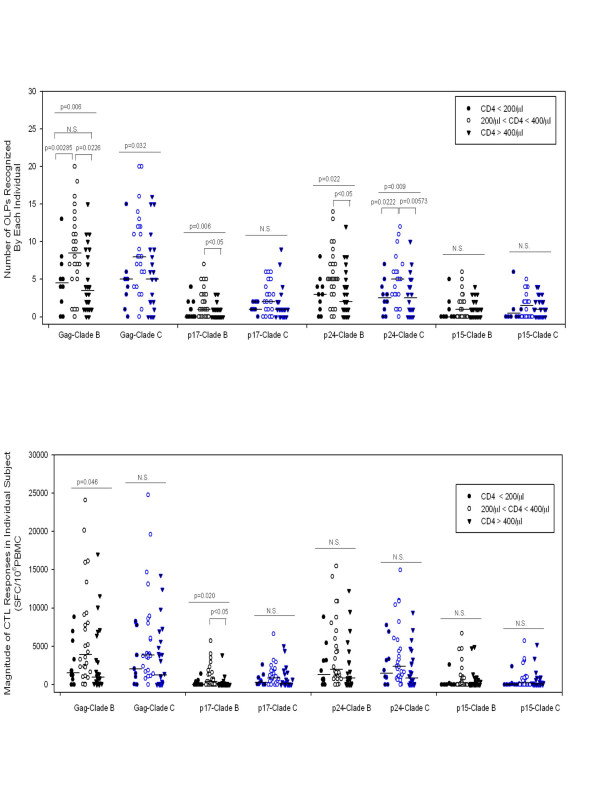
**Subjects grouped with different CD4 cell counts mounted a different magnitude and breadth of CTL responses targeting Gag**. For each portion of the HIV-1 proteins (Gag, p17, p24 or p15), the total magnitude or breadth of each individual is dotted and the median values are shown as a dash. Black dots are responses targeting consensus clade B peptides and blue dots for responses targeting clade C peptides. The filled dots designate the values from the group of CD4 cell counts less than 200/μl, the circles for individuals with CD4 cell counts ranging from 200–400/μl and the triangles for CD4 cell counts more than 400/μl. The p values were obtained using One Way Analysis of Variance (ANOVA) for multiple group comparison and Dunn's method or Holm-Sidak method for pair wise multiple comparison, when appropriate for data distribution.

Firstly, we examined the correlation of CTL responses with CD4 cell counts and viral loads and found that there are no significant correlations between the overall breadth of responses and the CD4 cell count or plasma viral load. However, a weak negative correlation between the total magnitude and the CD4 cell count was observed (R = -0.260, p = 0.0442 for clade B OLP set; R = -0.283, p = 0.0285 for clade C OLP set, Pearson Correlation test). When looking at specific HIV-1 proteins, we found that an increased breadth of CTL responses targeting Gag (especially p24 and p15) resulted in decreased plasma viral load, while for Nef and Vpu, increased breadth or magnitude of CTL responses corresponded to increased plasma viral load. However, these correlates between the breadth of CTL responses to specific proteins and the plasma viral load can only be observed in consensus clade C peptide sets, with the exception of p15. We then classified the subjects into three groups based on their CD4 cell counts and compared the breadth and magnitude of CTL responses. The results show stronger and broader CTL responses in subjects with CD4 cell counts ranging 200–400/mm^3 ^than those with less than 200/mm^3 ^or more than 400/mm^3^. By One Way Analysis of Variance (ANOVA), we find that there are significant differences between the three groups in CTL responses targeting clade B Gag (magnitude p = 0.046, breadth p = 0.006), clade B p17 (magnitude p = 0.020, breadth p = 0.027), and clade B p24 (breadth p = 0.022); clade C total breadth (p = 0.032), clade C gag (breadth p = 0.022), clade C gag-p24 (breadth p = 0.009), clade C Nef (breadth p = 0.045, magnitude p = 0.023), and clade C gp41 (magnitude p = 0.024). However, by pair wise multiple comparison, the differences with statistical significance are only observed in the breadth of clade B gag (200–400 vs. >400, unadjusted p = 0.00285; <200 vs. 200–400, unadjusted p = 0.0226), clade B gag-p17 (200–400 vs. >400, p < 0.05), clade B gag-p24 (200–400 vs. >400, p < 0.05), clade C gag-p24 (200–400 vs. >400, unadjusted p = 0.00573; <200 vs. 200–400, unadjusted p = 0.0222), clade C Nef (200–400 vs. >400, p < 0.05), and in magnitude of clade B Gag-p17 (200–400 vs. >400, p < 0.05), Clade C Nef (200–400 vs. >400, p < 0.05). Figure 4 shows the different magnitudes and breadths of CTL responses targeting Gag protein when the subjects were classified using their CD4 cell counts.

## Discussion

Several studies have been performed to characterize the immune responses of HIV-1 infected populations of Chinese origin [26-29]. However, these studies have focused on subjects infected with the clade B virus, which circulates throughout central China in former plasma donors. As shown in the two nationwide HIV-1 molecular epidemiological surveys performed in China in 1998 and 2004, the B'/C recombinant strains (particularly CRF07_BC) are circulating in western China from south to north [18-22]. This is the first study to address the profile of cellular immune responses in Chinese subjects infected with HIV-1 CRF07_BC.

It was reported that Gag, Pol and Nef are among the most frequently targeted proteins by CTL in subjects infected with HIV-1, including clades B and C [9,10,12,13,29,30]. Such clustering pattern of CTL epitopes in HIV-1 proteins has led to the postulate that the frequency of CTL recognition is inversely correlated with the variability of the viral sequences[31,32]. We have seen the similar clustering pattern of CTL responses targeting HIV-1 proteins in this study population infected with HIV-1 CRF07_BC. Namely, Gag, Pol and Nef are among the most frequently targeted proteins, while Vpu and Tat are rarely targeted (Figure 1, Table 1). Also, we have observed that Vpr and Vif are targeted intensively. However, Pol-PR may be exceptional to the postulate of inverse correlation between frequency of CTL recognition and variability of the viral sequences, as we have not observed immunodominant epitopes in this relatively conserved protein (Table 1, Figure 2).

The identification of immunodominant regions is crucial for vaccine development and evaluation as these are the targeted HIV regions that will be included in promising vaccines [15]. Immune escape from immunodominant epitopes can result in a broader spectrum of CTL responses and in a faster development to AIDS[1,33] and the host's genetic background may drive the elimination of subdominant yet effective epitopes from circulating viral population[34]. In this study, we identified 89 immunodominant OLPs scattering the HIV-1 proteins except for Pol-PR, Tat and Vpu (Figure 2). We also observed that the subdominant epitopes were also distributed throughout the HIV-1 expressed genome. When comparing the patterns of dominant and subdominant epitopes detected by clades B or C peptides, Pol and Vif are notable for their discrepancies. The factors contributing such differentially targeting of clades B and C OLPs by CTL responses could be the cumulative effect of immune escapes during the HIV-1 epidemic in this population or the founder effects of viral linage[35]. Anyway, our data suggest that when incorporated into a vaccine construct, Gag and Nef can more easily induce cross-clade CTL responses, while the CTL responses induced by Pol and Vif are more clade-specific.

High cross-clade CTL responses have previously been extensively explored, especially in populations infected with clade B[14,29,36]. Cross-clade CD8 T-cell responses to HIV-1 CRF07_BC circulating in China have been recorded in a previous study by using recombinant vaccinia viruses containing HIV-1 genes as stimulus antigen [37]. However, by studying cross-clade CTL responses on the single peptide level, new insight can be achieved, keeping in mind that the homologous peptides can detect CTL responses better than recombinant vaccinia viruses expressed antigen and heterogeneous peptides [38]. We have demonstrated here that for the B'/C recombinant HIV-1 infected subjects, high cross recognition of consensus clades B and C peptides is also evident. However, we noticed that the B'/C recombinant strains contained part of the clade B sequences in Gag, Pol, Env, Nef and accessory genes except for Vif [18,21]. The relatively stronger and broader responses directed to clade C peptides compared with clade B was consistent with the reports in other studies, which show that homologous peptides are better at detecting CTL responses [38] and different from the observation in another study on a Chinese population infected with HIV-1 Thai B [29]. In the study by Zhao S et al, they observed no significant differences between the CTL responses targeting clade B and C peptide sets[29]. This may indicate that the recombinant form of HIV-1 CRF07_BC displays subtle differences in inducing the host's immune responses. From the recognition patterns in the clades B and C proteome, we can find that Pol-PR and Vpu tend to be targeted in the clade B sequence rather than the clade C sequence. These data are in line with the recombinant pattern of CRF07_BC, the genome of which are inserted with fragments of clade B sequences in *pol-pr *and *vpu *[18,21,22].

In the past decade, the correlation between CTL responses and immune control of HIV-1 infection has been extensively explored and controversial results have been reported[5,8,13,28,39-42]. A recent study has demonstrated that CTL responses to different HIV proteins have discordant associations with plasma viral load, which results in effective CTL responses without a demonstrable biological impact in chronic HIV infection[7]. The association between the breadth of Gag-specific CTL responses and low viremia has been confirmed in several population based studies [6-8]. Consistent with these studies [7,8], we have observed no statistically significant correlation between total magnitude or breadth of CTL responses and plasma viral load or CD4 cell count in this study. But the data demonstrate that the relatively broader CTL responses targeting Gag (especially Gag-p24 and p15) correlate with lower plasma viral loads, and broader CTL responses targeting Nef and Vpu correlate with increased viral loads. The rationale behind this finding is still to be elucidated. However, there are two possibilities to explain the discordance. The first possibility is that Nef and Vpu-specific CD8+ T-cell responses are as effective as Gag-specific responses in controlling viral replication, but the CTL responses are recruited sequentially to different viral antigens and escaped by virus with mutations in CTL epitopes [1,43]. An alternative explanation is that Nef and Vpu-specific CTL responses are inherently less effective than Gag-specific responses, partly due to the deleterious effect of the viral mutation in CTL targeted Gag protein[44]. In line with this, while several vaccine approaches that focus primarily or exclusively upon generation of a CTL responses protected macaques from disease, previous evidence also suggests that CTL-based vaccines no matter raised against densely conserved coding regions of HIV-1 spaning open reading frames such as Env, Tat and Rev simultaneously, can apparently always create viral escapes which are not necessarily confer a fitness cost[45]. Put these together, final validation of vaccine concept of eliciting protective CTL responses against invading HIV-1 will have to be obtained from large-scale efficacy clinical trial with promising HIV vaccines containing different viral products. The fact that the correlates can only be observed when tested with consensus clade C peptides other than clade B peptides indicates that the choice of test peptide may have an impact on the demonstration of the correlation between the CTL responses and the containment of viral load.

The further analysis by grouping the research subjects on basis of CD4 cell count, show that subjects with CD4 cell ranging 200–400/mm^3 ^mounted stronger CTL responses than those with less than 200/mm^3 ^or more than 400/mm^3^. The results suggest that the correlation between HIV-specific CTL responses and viral load in HIV-1 infection is dependent on disease status, which have been recorded in previous reports that weaker anti-HIV CD8+ T-cell effector activity were observed in HIV primary infection compared with asymptomatic subjects with chronic infection [28,46]. The decline of the HIV-1 specific CTL responses late in disease progession is also obvious and can be explained by the progressive depletion of CD4 helper T cells, which result in the inability of the body to mount broader and stronger CD8 CTL response targeting viral proteins [47], or by selective depletion of virus specific CTL [48] and the impaired proliferative capability of virus specific CD8 CTL[49], which lead to decreased effector activity of previously induced CTL responses.

## Conclusion

Overall, this is the first study addressing the profile of immune responses in Chinese subjects infected with HIV-1 B'/C recombinants. We have found similar CTL response patterns as previous reports [9,10,12,13,29]. However, by comparing CTL responses targeting the clades B and C proteome in the same population, we find significant differences in the total magnitude and breadth conferred by Gag-p17, Pol, and Env. This indicates that the rapidly overspread CRF07_BC may have subtle differences in inducing a host's immune responses when compared with the HIV-1 Thai B viral strain circulating in central China.

## Methods

### Study population

Sixty IDUs infected with HIV-1 CRF07_BC were recruited from Urumuqi at Xinjiang Uyghur Autonomous Region, which is located in northwestern China. The clinical and demographic characteristics of these subjects were as follows: median age, 32 years (range, 23–47 years); median HIV-1 RNA, 21,550 copies/ml plasma (range, 49–650,000 copies/ml plasma); median CD4 cell count, 339 cells/mm3 (range, 16–940 cells/mm3). All individuals were anti-retroviral therapy naive at the time of study and infected with HIV-1 CRF07_BC. The study was approved by the institutional review board of National Center for AIDS/STD Control and Prevention (NCAIDS, China-CDC) and was conducted in accordance with human experimentation guidelines.

### Synthetic HIV-1-peptides

Four-hundred and thirteen synthetic 15–20 amino acid long peptides, overlapping by 10 amino acids and spanning the entire HIV-1 clade B or C consensus sequence [50], were synthesized at the Massachusetts General Hospital (MGH) Peptide Core Facility on an automated peptide synthesizer using Fmoc technology. All peptides were synthesized at the same time and using the same reagents. Except for a few cases of insertion or residue deletions between clades, corresponding peptides from the different consensus sequences were always of the same length and spanned identical regions.

### Elispot assays

Elispot assays were carried out as described previously [30]. Briefly, peripheral blood mononuclear cells (PBMC) isolated by Ficoll-paque™ Plus (Amersham Biosciences) density gradient centrifugation were plated in 96-well polyvinylidene plates that had been precoated with 100 μl of anti-human interferon-gamma monoclonal antibody (0.5 μg/ml, Mabtech, Stockholm, Sweden). PBMCs were plated at a concentration of 100000 cells/well in a volume of 100 μl of RPMI 1640 medium supplemented with fetal calf serum (10%), Hepes buffer (10 mM), L-glutamine (2 mM) and penicillin-streptomycin (50 U/ml). Corresponding clades B and C peptides were combined into pools of four to six peptides and tested individually when a peptide pool gave a positive response. The final concentration of the peptides in each well was 10 μg/ml. Plates were incubated overnight at 37°C, 5% CO_2 _and developed the next day as described elsewhere [30]. Wells containing PBMC and medium with PMA/Ionomycin or without any peptide were used as positive or negative controls, respectively, and run in triplicate on each plate. To calculate the number of specific T cell responses, the number of spots in the negative control wells was subtracted from the counted number of spots in each well. Responses were considered positive if there were > 50 spot-forming cells (SFC)/1 × 10^6 ^PBMC after subtracting background and at least three times the mean number of SFC of the three control wells.

### Intracellular Cytokine Assay

ICS for IFN-gamma was performed as previously described [51]. 1 × 10^6 ^PBMC were incubated with peptide pools of 2 μg/ml Env, Gag, Pol, Nef and VVVRT(Vif, Vpr, Vpu, Rev and Tat) along with anti-CD28 and anti-CD49 antibodies (BD Pharmingen) at 37°C and 5% CO_2 _for 1 hour before the addition of brefeldin A (10 μg/ml; Sigma). The cells were incubated for an additional 5 hours at 37°C and 5% CO_2 _and then washed and stained with anti-CD4-PE and anti-CD8-APC antibodies (BD Pharmingen) at 4°C for 30 min. The cells were fixed with solution A (Caltag), permeabilized with solution B (Caltag), and then stained with fluorescein isothiocyanate-conjugated anti-IFN-gamma antibody. Flow cytometric analysis was performed on FACSCalibur with CellQuest Pro (Becton Dickinson). The FCS data were analyzed with FlowJo software.

### Statistical analysis

Results are given as means +/- SD or medians with ranges. Statistical analysis was performed with SigmaPlot version 10.0 (Systat Software, Inc.) and based on Student t tests, a Wilcoxon rank sum test, or a multiparametric ANOVA test, as appropriate; a P < 0.05 was considered significant. Viral-load values below the limit of detection of 50 RNA copies/ml were assigned a value of 49 for statistical analyses.

## Competing interests

The author(s) declare that they have no competing interests.

## Authors' contributions

YZ, YR recruited the subjects and collected the samples. JC, KH, XZ, HZ carried out the ELISPOT assays, HL, MJ, SL carrried out the ICS assays, HP, PM, HX performed the CD4 cell count and viral load tests. JC and KH carried out the data analysis and drafted the manuscript. XGY, MA, participated in the design of the study and coordination. KLW participated in the data analysis and helped to draft the manuscript. BDW, YS conceived of the study, and participated in its design and coordination. All authors read and approved the final manuscript.
